# Isolation of a SARS-CoV-2 strain from pediatric patients in South Korea: biologic and genetic characterization

**DOI:** 10.3389/fmicb.2025.1654224

**Published:** 2025-08-26

**Authors:** Hee Chun Chung, Sung Jae Kim, Su Jin Hwang, Sung Hoon Park, Kyoung Min Park, Hyeon Woo Chung, Si Hwan Ko, Dong il Park, Jun-Yeop Shim, Van Giap Nguyen, Jae Myun Lee

**Affiliations:** ^1^Department of Microbiology and Immunology, Institute for Immunology and Immunological Diseases, Yonsei University College of Medicine, Seoul, Republic of Korea; ^2^Department of Companion Animal Health, Kyungbok University, Namyangju, Republic of Korea; ^3^Department of Microbiology and Immunology, Institute for Immunology and Immunological Diseases, Brain Korea 21 Project for Medical Science, Yonsei University College of Medicine, Seoul, Republic of Korea; ^4^R&F Chemical Co., Ltd, Hanam, Gyeonggi, Republic of Korea; ^5^Department of Veterinary Microbiology-Infectious Diseases, Faculty of Veterinary Medicine, Vietnam National University of Agriculture, Hanoi, Vietnam

**Keywords:** SARS-CoV-2, pediatric patient, biology, genetic characterization, N15 strain

## Abstract

**Introduction:**

Pediatric isolates of SARS-CoV-2 remain underrepresented in virologic studies, despite their importance for understanding viral diversity and therapeutic responses.

**Methods:**

Nasal swab and saliva samples were collected from pediatric COVID-19 patients. Viral isolation was attempted in Vero cells through five blind passages. Replication was assessed by digital RT-PCR, while cytopathic effects were observed microscopically. Genomic sequencing was conducted using next-generation sequencing, and antiviral activity was evaluated for Remdesivir, Molnupiravir, and Nirmatrelvir.

**Results:**

A SARS-CoV-2 strain, designated N15, was successfully isolated from a pediatric nasal swab. The isolate replicated efficiently in Vero cells with kinetics comparable to B.1 and B.1.1.529 lineages. Cytopathic effects appeared within 48 h post-infection, marked by aggregates of dead cells. Genomic analysis classified N15 within lineage 19B, showing 99.9% similarity to the Wuhan-Hu-1 strain. Rare mutations were identified: N709S in the spike protein and T11M in the E protein. Antiviral testing revealed effective inhibition by Remdesivir, Molnupiravir, and Nirmatrelvir, with varying IC50 values across cell types.

**Discussion:**

The pediatric N15 isolate represents a unique 19B lineage virus, retaining ancestral genomic features while harboring rare mutations. Its efficient replication and drug sensitivity underscore its value as a reference strain for comparative studies against circulating variants and for evaluating antiviral efficacy.

## 1 Introduction

Among the seven human coronaviruses known to date, three lethal viruses have emerged in the past two decades: severe acute respiratory syndrome coronavirus (SARS-CoV) in 2003, Middle East respiratory syndrome coronavirus (MERS-CoV) in 2012, and the most recent Coronavirus Disease 2019 (COVID-19) pandemic caused by SARS-CoV-2 (formerly 2019-nCoV) (Chen et al., 2020; Coronaviridae Study Group of the International Committee on Taxonomy of Viruses, 2020). Regarding genomic characteristics, the genome of SARS-CoV-2 has been determined to contain 29,829 nucleotides in length (Lu et al., 2020) and possesses 14 open reading frames (ORFs) in order of ORF1ab, ORF1a, spike (S), 3a, 3b, envelope (E), matrix (M), p6, 7a, 7b, 8b, 9b, nucleocapsid (N), and ORF14 genes (Wu et al., 2020). Though belonging to the same genus *Betacoronavirus*, subgenus *Sarbecovirus* (Coronaviridae Study Group of the International Committee on Taxonomy of Viruses, 2020), SARS-CoV-2 differs from its sister virus SARS-CoV by 380 amino acid substitutions in 9 of the 14 genes (Wu et al., 2020) and bears a furin-like cleavage site (RRAR) insertion at the S1/S2 junction (Goldsmith et al., 2004; Harcourt et al., 2020). Notably, SARS-CoV-2 continues to evolve, generating variants (Dubey et al., 2021) that, over time are gradually replacing older variants (Beesley et al., 2023). Recent studies have provided comprehensive insights into its genomic architecture and evolutionary dynamics, highlighting mechanisms of genetic variation and implications for therapeutic development (Bhardwaj et al., 2023; Zabidi et al., 2023; Erkihun et al., 2024). Additionally, different systems have been developed to track this rapidly diversifying virus, such as GISAID, NextStrain, and PANGOLIN (Abulsoud et al., 2023). Some of these variants alter the virus’s properties and may be further defined by WHO as variants of concern (VOCs) and variants of interest (VOIs) (Konings et al., 2021).

In South Korea, following the first case of COVID-19 on 20 January 2020, studies have encompassed various topics, such as epidemiology, pathology, prevention, and control (Ki and Task Force for 2019-nCoV, 2020; Shim et al., 2020). Among these, the genomic epidemiology of SARS-CoV-2 variants has emerged as an active field. It has been revealed that circulating SARS-CoV-2 variants has created different waves, each wave dominated by specific variants (Kim et al., 2023; No et al., 2024). However, we describe here a rare pediatric N15 isolate collected during a transitional phase of lineage A circulation. Its uncommon mutations and retained ancestral genomic features, together with its limited spread, offer valuable clues to the genetic constraints on SARS-CoV-2 evolution. In addition to genomic characterization, the study examines biological properties, including *in vitro* replication and susceptibility to selected antiviral agents, to compare with other lineages and assess whether its unique genomic profile is linked to changes in growth or drug response.

## 2 Materials and methods

### 2.1 Sample collection

From May to November 2021, we collected 15 nasal swab samples and six saliva samples from 16 pediatric patients who exhibited clinical symptoms and had high SARS-CoV-2 viral titers as detected in our previous study (Kim et al., 2024) on the development of a new diagnostic method using the Proteinase K method for SARS-CoV-2 detection. The samples were collected at Inha University Hospital (Incheon, Korea) under protocol number 2021-07-008. These collected samples (Table 1), which were stored at −80 °C, were sent to the College of Medicine at Yonsei University. The handling and processing of these samples were conducted at Biosafety Level 3 (BSL3) in the Avison Biomedical Research Center, with the approval of the Institutional Biosafety Committee (IBC 2022-0320).

**TABLE 1:** Sampling information of SARS-CoV-2 positive pediatric samples.

Sample number	Sample name	Collection date (year-month-date)	Sex	Age	Sample type	Clinical symptoms	Ct value for SARS-CoV-2 detection
N1	JHE	2021-05-16	Female	4.7	Nasal swab	Fever	19.87
N2	LYJ	2021-05-16	Male	7.1	Nasal swab	Fever, respiratory problems	18.08
N3	LMC	2021-06-02	Male	6.0	Nasal swab	Fever, sore throat, respiratory problems	20.96
N4	KHL	2021-06-30	Male	8.4	Nasal swab	Fever, respiratory problems	18.20 24.84
N5					Saliva		
N6	KHR	2021-07-07	Female	12.4	Saliva	Fever, sore throat, respiratory problems	23.12 23.95
N7					Nasal swab		
N8	JYA	2021-08-21	Female	8.4	Nasal swab	Fever	20.13 20.36
N9					Saliva		
N10	SSK	2021-09-03	Male	11.2	Nasal swab	Fever, respiratory problems	20.03
N11	LUB	2021-07-08	Male	13.9	Nasal swab	Fever, sore throat, respiratory problems	25.52 24.12
N12					Saliva		
N13	LGE	2021-07-08	Female	11.7	Saliva	Fever	23.19 25.95
N14					Nasal swab		
N15	SSM	2021-09-19	Female	12.3	Nasal swab	Fever, Phlegm	21.62
N16	LIY	2021-10-29	Female	13.0	Nasal swab	Fever, Sore throat,	17.90
N17	KJS	2021-10-21	Male	7.9	Nasal swab	Fever, Headache	19.34
N18	KJH	2021-10-21	Male	9.0	Nasal swab	Fever	22.42
N19	KJB	2021-11-01	Male	14.7	Nasal swab	Fever	20.57
N20	CLA	2021-09-17	Female	7.3	Nasal swab	Fever, respiratory problems, sense of confinement	26.76
N21	LAR	2021-08-08	Female	12.0	Saliva	Fever	27.71

### 2.2 Isolation of SARS-CoV-2 in Vero cell line

Attempts were made to isolate SARS-CoV-2 from pediatric samples using Vero cell lines (ATCC No. CCL-81). An overnight monolayer of Vero cells (80% confluence) was washed twice with 1 × phosphate-buffered saline (PBS) before inoculating the nasal and saliva samples, which were filtered through a 0.2 μm filter, at a diluted to 5% (w/v) in Dulbecco’s Modified Eagle Medium (DMEM) containing antibiotics prior to inoculation. After 1 h of adsorption at 37 °C with 5% CO_2_, a maintenance medium was added. This medium consisted of Dulbecco’s Modified Eagle Medium (DMEM) supplemented with trypsin (10 μg/mL), yeast extract (0.04%), tryptose phosphate broth (0.6%), and Antibiotic-Antimycotic 100 × (4 μL/mL; Gibco, Thermo Fisher Scientific, Grand Island, NY, United States) at a ratio of 1:10. The inoculated cells were cultured for 3–4 days at 37 °C in a 5% CO_2_ atmosphere and were blindly passaged five times. Among the pediatric nasal swab and saliva samples of SARS-CoV-2, one strain, named N15, was successfully isolated in Vero cells. All fifth blind passage samples were titrated for SARS-CoV-2 using digital RT-PCR (dRT-PCR).

### 2.3 Digital RT-PCR based for SARS-CoV-2 confirmation

Total RNA was extracted from the fifth passage using the RNeasy Mini Kit (Qiagen, Valencia, CA, United States), following the manufacturer’s instructions. The reaction mixture of 30 μl included 5 μl of primer–probe mixture (Supplementary Table 1; 20 pmol forward primer, 20 pmol reverse primer, and 10 pmol FAM-BHQ1 probe per reaction), 15 μl of 2X Dr. PCR OneStep dRT-PCR Mixture (Optolane, Lot 4M009B-2201), 5 μl of total RNA, and nuclease-free water up to 30 μl. The reaction mixtures were loaded into wells of LOAA Dr. Digital PCR cartridges (Optolane, Seongnam-si, Korea). The cartridges were placed into the POSTMAN equipment (Optolane, Seongnam-si, Korea) for uniform application, and then mounted on the LOAA equipment (Optolane, Seongnam-si, Korea). The thermal profile began with an incubation at 50 °C for 30 min to convert RNA into cDNA, followed by denaturation at 95 °C for 15 min. The subsequent cycling conditions for digital PCR included 40 cycles of 95 °C for 10 s and 60 °C for 15 s. Each sample produced 16,800 to 19,200 valid wells. Digital PCR results were analyzed using the “Optolane OnPoint Pro” software (Optolane, Korea).

### 2.4 Immunofluorescence assay (IFA) to confirm the replication of SARS-CoV-2

The passage five of the N15 strain was inoculated into fresh Vero cells at a multiplicity of infection (MOI). After an absorption period of 1 h at 37 °C, the cells were washed twice with 1 × PBS and then cultured in the maintenance medium for 24 h (subsection 2.2). Following the 24 h incubation, the Vero cells were fixed with 4% formaldehyde, permeabilized with 0.1% Triton X-100 in PBS, and blocked with 5% bovine serum albumin in PBS for 1 h. Primary rabbit monoclonal antibodies against SARS-CoV-2 nucleocapsid protein (Abcam, cat. no. ab271180) were added to the cells and incubated at room temperature for 1 h. After washing, a fluorescently conjugated secondary antibody, Alexa Fluor 647-labeled goat anti-rabbit IgG (red), was added and incubated at room temperature for 2 h. The cell nuclei were stained with one drop of VECTASHIELD Antifade Mounting Medium with DAPI (Vector Laboratories, Burlingame, CA, United States) and allowed to dry. The images were analyzed using a FluoView FV1000 confocal microscope (Olympus Life Science, Tokyo, Japan).

### 2.5 Electron microscopy to confirm the presence SARS-CoV-2

Viral supernatant from passage level 5 was inoculated at a 0.1 MOI into confluent Vero cells in a 25T flask and incubated for 72 h. To observe the virus using a transmission electron microscope (TEM), the viral-inoculated Vero cells from the 25T flask were harvested at 2,500 rpm at 4 °C for 10 min. Subsequently, the cells were washed and dehydrated using a graded ethanol series (50%, 60%, 70%, 80%, 90%, 100%). The dehydrated cells were infiltrated with an Embed-812 embedding kit (Electron Microscopy Sciences, United States). The embedding was polymerized in an oven at 60 °C for 48 h. Finally, the samples were negatively stained and examined using an HT7800 transmission electron microscope (Hitachi High-Tech Corporation, Japan).

### 2.6 SARS-CoV-2 titration by plaque assay

The supernatants of passage 5 and 8 of the N15 strain were harvested and subjected to 10-fold serial dilutions (10^–2^ to 10^–6^). Each dilution (0.25 mL) was then added in duplicate to wells of confluent Vero cells. After 1 h absorption period, the inoculum was discarded, and each well was rinsed with PBS. The cells were then overlaid with 1% low melting temperature agarose containing DMEM, 2% fetal bovine serum, and 1% penicillin/streptomycin. After 48 h of incubation at 37 °C, the plaques were stained using a Neutral Red Solution (0.33% w/v in DPBS; Sigma, Cat. No. N2889). After an additional 24 h of incubation, the number of plaques (N) was counted. The following formula was applied to determine the titer of viral stock: PFU/mL = N/(D × V), where D is the dilution factor, and V is the volume of diluted virus added per well.

### 2.7 Viral growth kinetics in Vero cells

The replication kinetics of the N15 strain were assessed and compared with those of two other SARS-CoV-2 lineages obtained from the Korean National Culture Collection for Pathogens: B.1 (NCCP-43344) and B.1.1.529 (NCCP-43408). The viruses were inoculated onto Vero cell monolayers at a multiplicity of infection (MOI) of 0.1. Virus adsorption was carried out for 1 h at 37 °C. Following adsorption, the cells were washed twice with phosphate-buffered saline (PBS) to remove unbound virus particles and then incubated in maintenance medium for up to 120 h. The maintenance medium consisted of Dulbecco’s Modified Eagle Medium (DMEM) supplemented with 0.3% tryptose phosphate broth, 0.02% yeast extract, and 1 μg/mL trypsin. Supernatants were collected at 1-, 24-, 48-, 72-, 96-, and 120 h post-inoculation (hpi) to monitor viral replication. Viral RNA was extracted and quantified using dRT-PCR targeting the nucleocapsid (N) gene (Supplementary Table 1). All samples were analyzed in triplicate (see section “2.3 Digital RT-PCR based for SARS-CoV-2 confirmation”).

### 2.8 Genomic characterization of a SARS-CoV-2 isolate

Total RNA of the N15 strain was extracted using the RNeasy mini kit (Qiagen, Valencia, CA, United States) and subsequently sent for next-generation sequencing at Macrogen (Korea). The library was constructed using the TruSeq Stranded Total RNA Library Prep Gold Kit, and sequencing was performed on the Illumina NovaSeq 6000 platform. A total of 49,285,634 reads were produced for the N15 sample, with a GC content of 39.8% and a Q30 score of 94.0%. For bioinformatic analysis, the reads were processed using the Snakelines (v.1.18) framework (Goga et al., 2021). The complete genomes of the N15 strain have been deposited into the NCBI GenBank with accession number PP915627. Genomic annotation of the N15 strain was performed using GATU (Tcherepanov et al., 2006) with the reference genome of the Wuhan-Hu-1 isolate (NC_045512.2).

For genetic classification, various levels of analysis were applied in this study. The first was based on genome sequences using Nextclade (Aksamentov et al., 2021) and Pangolin tool (O’toole et al., 2021). The second one based on the spike sequence using Hedgehog tool (O’toole et al., 2022). To determine the fluctuation over time of different lineages of SARS-CoV-2 in South Korea, complete genomic sequences of South Korea strains with collection dates (*n* = 192,501) were retrieved from GISAID (Supplementary Table 2). Spike sequences were extracted using the tool available at https://mafft.cbrc.jp/alignment/server/specificregion-last.html (last accessed September 2024). All 192,501 Korean spike sequences were then classified using the Hedgehog tool, resulting in 5,687 sequences belonging to lineage A_1 and 247 sequences belonging to lineage A_2 (Supplementary Table 2). The chronological frequency plot of lineages A_1 and A_2 of SARS-CoV-2 was generated using Microsoft Excel.

### 2.9 Antiviral assay of a SARS-CoV-2 isolate

The isolated N15 strain was subjected to an antiviral assay against three drugs known to inhibit coronaviruses (Tiseo et al., 2023): Remdesivir (Cat. no. HY-104077), Molnupiravir (Cat. no. HY-135853), and Nirmatrelvir (Cat. no. HY-138687), all of which were purchased from MedChemExpress.^1^ Vero and Calu-3 cells (6.25 × 10^4^ cells) were plated in a 48-well plate, cultured overnight, and pre-treated for 2 h with serial 2-fold dilutions of each drug at varying concentrations in triplicate experiments. The concentration range (μM) for Remdesivir were 10–0.156, Molnupiravir were 100–0.781, and Nirmatrelvir were 10–0.078. The cells were then inoculated with passage 5 of the N15 strain at a MOI of 0.01 for 1 h. After 1 h of inoculation, the virus was removed, the cells were washed once with PBS, and fresh medium containing dilutions of each drug was added to the cells. At 24 and 48 hpi, culture supernatants were harvested, and viral RNA was extracted for quantification of viral genomic copies by real-time RT-PCR. The half-maximal inhibitory concentration (IC_50_) values were calculated from the normalized activity dataset-fitted curves using GraphPad Prism v.10.2.3.

## 3 Results

### 3.1 Isolation of SARS-CoV-2 N15 strain using Vero cells

**FIGURE 1:**
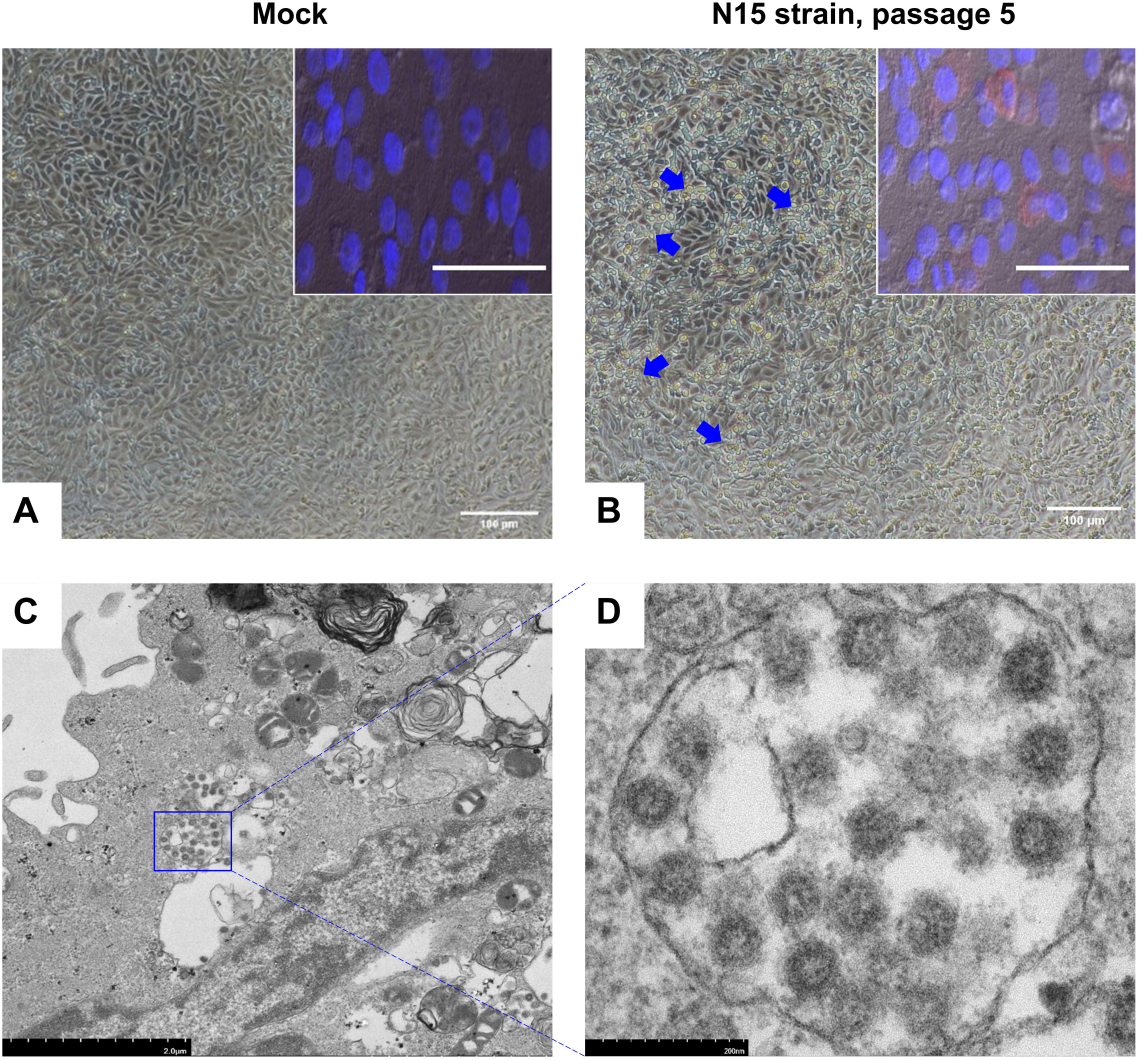
Images of Vero cells infected with the N15 strain at passage 5, 48 h post-infection (hpi). The mock group presents the negative control condition **(A)**. Blue arrows indicate changes in the infected Vero cells **(B)**. Insets show immunofluorescence images from an IFA analysis of the mock group and N15 strain at passage 5. The merged image combines staining signals of the nucleocapsid protein (red fluorescence) and cell nuclei (DAPI) for both the mock (inset A) and N15 strain at passage 5 (inset B). White scale bars at the bottom of each image represent 100 microns. Electron microscopy images of N15-infected cells reveal clusters of viral particles within membrane-bound vesicles in the cytoplasm **(C)**. A closer view of individual virus particles **(D)** shows their characteristic spherical shape and surface features. The scale bars in each image indicate the magnification levels.

The N15 strain of SARS-CoV-2 was successfully isolated in Vero cells after five blind passages. In Vero cells, cytopathic effects (CPE) were observed within 48 hpi, characterized by the cells beginning to detach from the monolayer and cluster into grape-like aggregates (blue arrows, Figure 1B). Besides of CPE, the replication of virus was confirmed through IFA and TEM. The IFA results demonstrated that the N15 strain at passage 5 exhibited strong staining for the nucleocapsid protein, which localized in the cytoplasm of Vero cells (bright red signal, inserted Figure 1B). These findings suggested that Vero cells had been productively infected by the N15 strain. The TEM results, shown in Figure 1C, reveal clusters of viral particles observed within membrane-bound vesicles in the cytoplasm. Figure 1D clearly illustrates the characteristic spherical shape and surface features of individual viral particles, measuring approximately 75 nm in size.

### 3.2 Titration of SARS-CoV-2 N15 strain passages 5 and 8 in Vero cells

**FIGURE 2:**
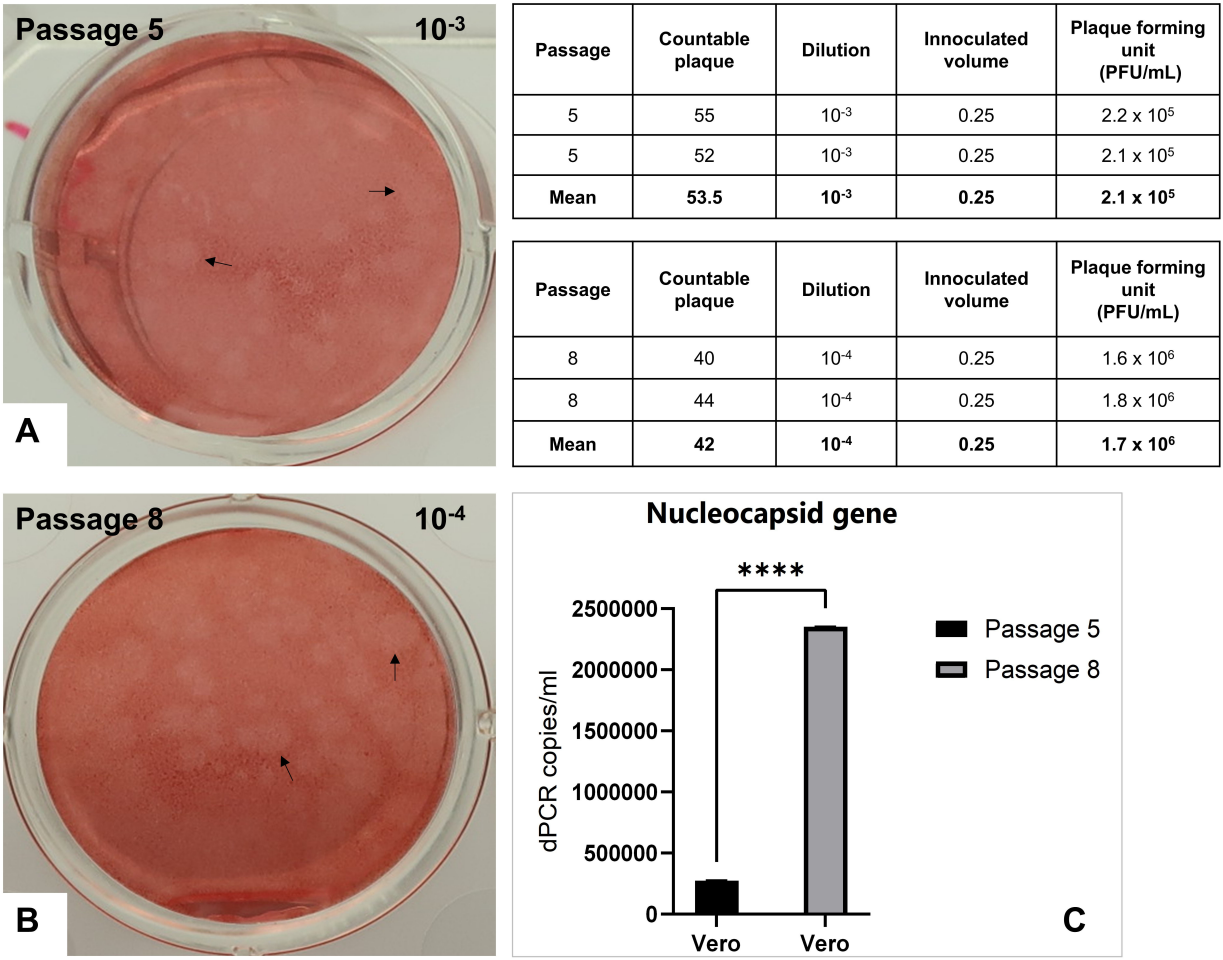
Titration of the N15 isolate by plaque assay. Images show plaques as large, light pink-colored circles formed on a monolayer of Vero cells at passage 5 with a 1,000-fold dilution **(A)** and at passage 8 with a 10,000-fold dilution **(B)**. Inserted tables provide the number of countable plaques at each corresponding dilution and the titer calculations. **(C)** Expression levels of the nucleocapsid gene for passages 5 and 8 were also evaluated by dRT-PCR. Statistical analyses were performed using GraphPad Prism v.10.2.3, with significant differences between passage 5 and passage 8 groups determined by paired *t*-test (****p < 0.001).

The N15 strain from both passage 5 and passage 8 produced large, light pink- colored circles on a red monolayer of cells (arrows, Figures 2A, B). The plaque assays revealed a significant increase in viral titers between passage 5 and passage 8. Specifically, the mean PFU/mL for passage 5 was 2.1 × 10^5^, whereas for passage 8, the mean PFU/mL increased to 1.7 × 10^6^. This indicates a considerable enhancement in viral replication or infectivity across the passages. The viral load in Vero cells increased significantly from passage 5 (mean 273,319 copies/ml) to passage 8 (mean 2,350,706 copies/ml) (Figure 2C). The higher expression levels of the nucleocapsid gene at passage 8 align with the increased viral titers observed in the plaque assay results.

### 3.3 Growth kinetics of SARS-CoV-2 strains in Vero cells

**FIGURE 3:**
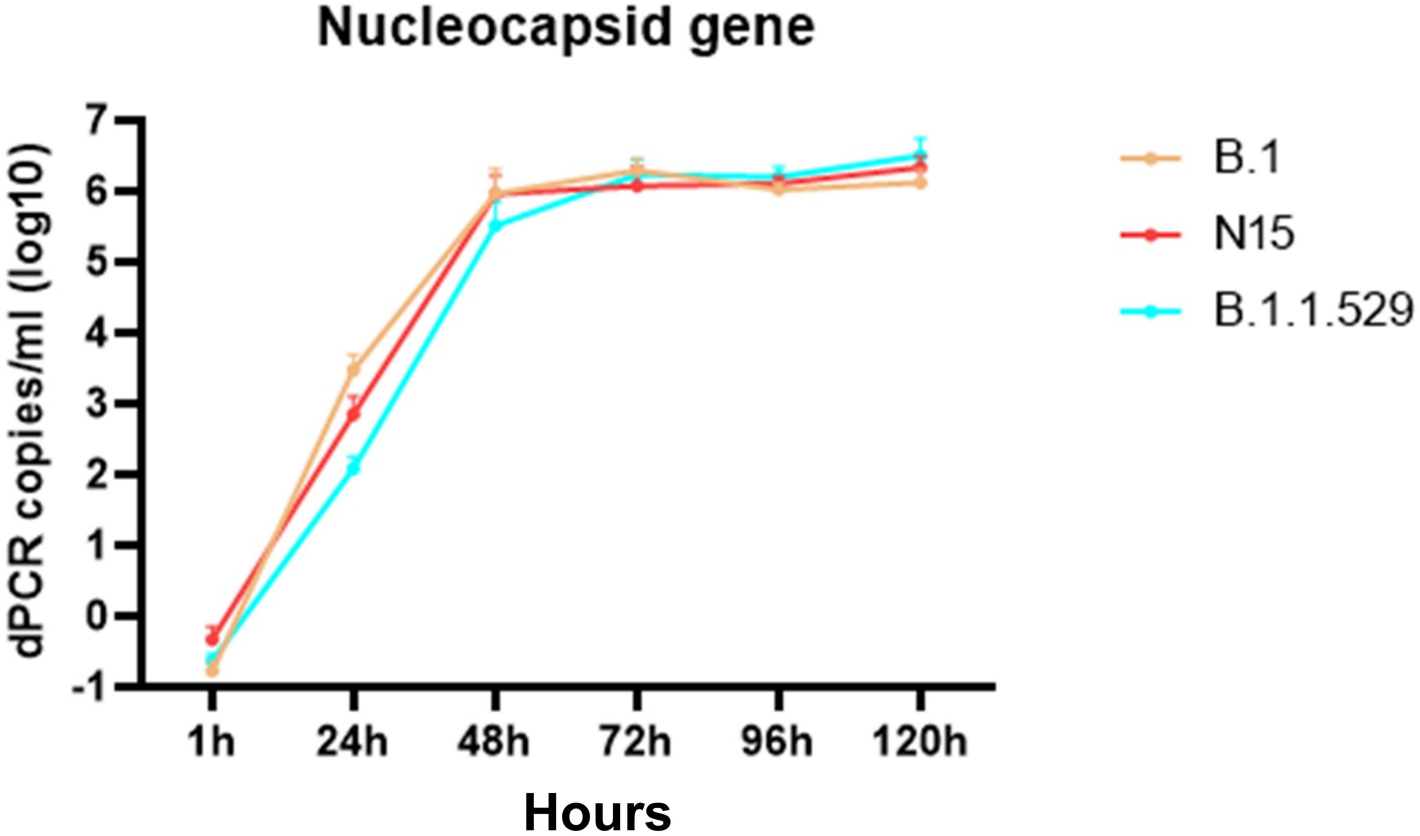
Time-dependent expression of the nucleocapsid gene in Vero cells infected with SARS-CoV-2 variants B.1, N15, and B.1.1.529. The figure shows that the expression levels of the nucleocapsid gene increased over time at 1, 24, 48-, 72-, 96-, and 120 h post-infection in Vero cells.

Because SARS-CoV-2 does not induce CPE in certain cell lines, the replication of three viral strains (B.1, N15, and B.1.1.529) was assessed in Vero cells by quantifying nucleocapsid gene expression using dRT-PCR. As shown in Figure 3, all three strains exhibited a marked increase in viral RNA levels over time. Viral loads increased rapidly from 1 to 48 h post-infection (hpi), reaching approximately 6 log_10_ copies/mL. After 48 hpi, the viral RNA levels plateaued, with only minor differences observed among the strains. Notably, all three strains reached similar nucleocapsid gene expression levels by 72 h post-infection (hpi) and remained stable up to 120 hpi. These results indicate that all tested strains replicate efficiently in Vero cells with comparable kinetics.

### 3.4 Genomic characterization of SARS-CoV-2 N15 strain

The genome of the N15 strain contained 29,893 nucleotides and exhibited a high similarity of 99.9% to the canonical SARS-CoV-2 reference genome (NC_045512.2). According to this reference, the N15 strain was predicted to encode 12 open reading frames (ORFs). Eight non-synonymous substitutions were observed in five out of 12 ORFs (Table 2). Among these mutations of N709S in the spike protein and T11M in the E protein were considered rare (based on information from CoVsurver^2^). Of the 23 sites in the nsp5 and 11 sites in the nsp12 protein associated with antiviral drug resistance (European Centre for Disease Prevention and Control, 2023; Flynn et al., 2023), no mutations were observed in the N15 strain, as well as in the other Korean strains of Pango lineage A found in different years from 2020 to 2023 (not shown).

**TABLE 2:** Genome annotation of N15 strain.

No.	Gene/ORF	Mature peptide	Start	Stop	Nucleotide mutation^$^	Amino acid mutation
1	1ab	Leader protein (nsp1)*	256	795	A527C	E91A
		nsp2*	796	2,709	–	–
		nsp3*	2,710	8,544	–	–
		nsp4*	8,545	10,044	–	–
		3C-like proteinase (nsp5)*	10,045	10,962	–	–
		nsp6*	10,963	11,832	–	–
		nsp7*	11,833	12,081	–	–
		nsp8*	12,082	12,675	–	–
		nsp9*	12,676	13,014	–	–
		nsp10*	13,015	13,431	–	–
		RNA-dependent RNA polymerase (nsp12)	13432.13458	13458. 16226	–	–
		Helicase (nsp13)	16,227	18,029	C16648T, C17094T	T1064I, H1213Y
		3’-to-5’ exonuclease (nsp14)	18,030	19610	–	–
		endoRNAse (nsp15)	19,611	20,648	–	–
		2’-O-ribose methyltransferase (nsp16)	20,649	21,542	–	–
2	1a	leader protein (nsp1)*	256	795	–	–
		nsp2*	796	2,709	–	–
		nsp3*	2,710	8,544	–	–
		nsp4*	8,545	10,044	–	–
		3C-like proteinase (nsp5)*	10,045	10,962	–	–
		nsp6*	10,963	11,832	–	–
		nsp7*	11,833	12,081	–	–
		nsp8*	12,082	12,675	–	–
		nsp9*	12,676	13,014	–	–
		nsp10*	13,015	13,431	–	–
		nsp11	13,432	13,470	–	–
3	S	Surface glycoprotein	21,553	25,374	A23678G G25012T	N709S^#^ E1150D
4	3a	ORF3a protein	25,383	26210	G26167T	V259L
5	E	Envelope protein	26,235	26,462	C26266T	T11M^#^
6	M	Membrane glycoprotein	26,513	27,181	–	–
7	6	ORF6 protein	27,192	27,377	–	–
8	7a	ORF7a protein	27,384	27,749	–	–
9	7b	ORF7b	27,746	27,877	–	–
10	8	ORF8 protein	27,884	28,249	T28134C	L84S
11	N	Nucleocapsid phosphoprotein	28,264	29,523	–	–
12	10	ORF10 protein	29,548	29,664	–	–

*Mature peptide produced by both ORF1ab and ORF1a. Start and stop nucleotide positions were based on N15 strain (PP915627). ^$^Nucleotide and amino acid mutation compared to reference Wuhan-Hu-1 isolate (NC_045512.2). ^#^Rare mutation of SARS-CoV-2 based on the information from CoVsurver^2^.

**FIGURE 4:**
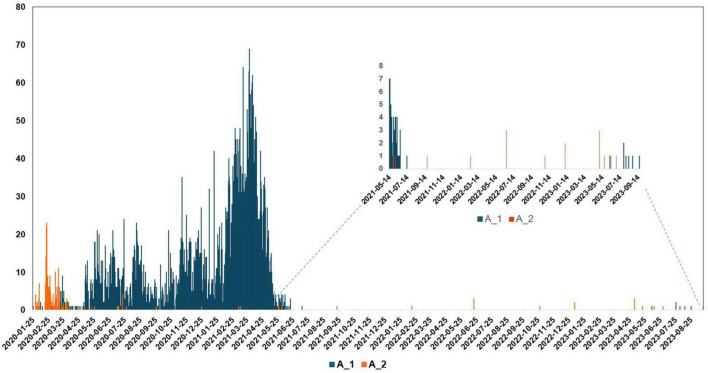
Lineage frequencies of SARS-CoV-2 over time in South Korea, focusing on lineages A_1 and A_2. Lineage A circulated primarily during the early period of the pandemic in South Korea. After July 2021, it was detected only occasionally, but it persisted at low levels until at least July 2023. The x-axis represents the collection date, while the y-axis shows the number of sequences.

Based on genome classification, the N15 strain belongs to Pango lineage A and Nextstrain clade 19B (Supplementary Figure 1). Based on the spike protein sequence, the N15 was classified as A_2 lineage. Unlike most globally circulating SARS-CoV-2 variants that possess the D614G mutation (O’toole et al., 2022), the N15 spike retains the ancestral D (aspartic acid) at position 614. The absence of the D614G mutation further supports its classification within the early A_2 lineage. In the epidemiological context, the unique situation of lineage A in South Korea is illustrated in the accompanying Figure 4. From January 2020 to July 2023, it was observed that lineage A predominantly circulated during the initial phase of the pandemic. After July 2021, sequences of lineage A were detected only sporadically; however, this lineage continued to persist at least until July 2023. The N15 strain was collected in September 2021, following a decline in the epidemic wave of lineage A.

### 3.5 Antiviral susceptibility of SARS-CoV-2 N15 strain

**FIGURE 5:**
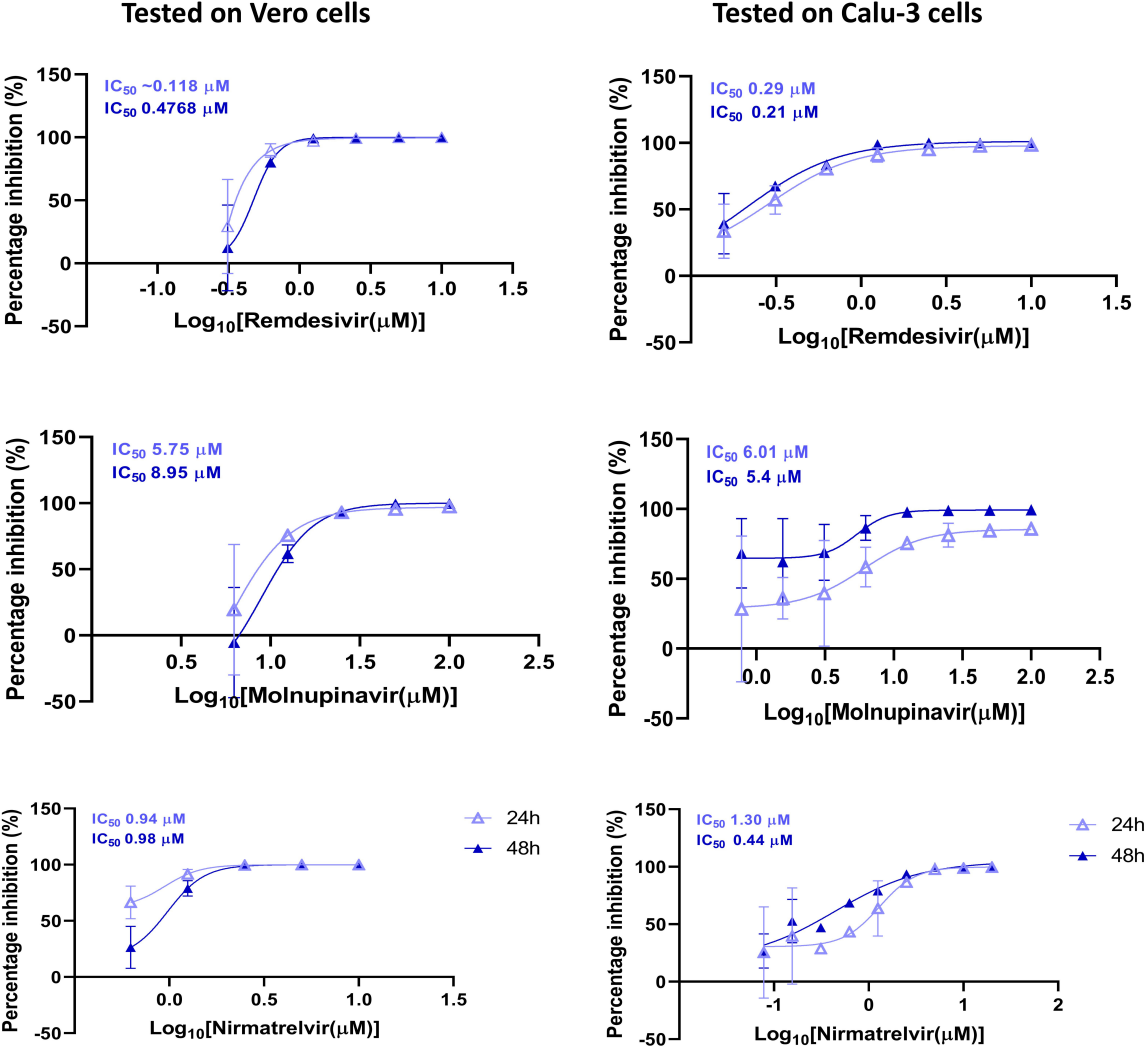
Antiviral susceptibility of the SARS-CoV-2 N15 strain. The graphs show the percentage inhibition of viral replication at various drug concentrations in Vero and Calu-3 cell lines, measured at 24 and 48 hpi. The IC_50_ values for each drug are displayed at the top of the respective y-axis in each graph.

In Figure 5, the antiviral efficacy of three drugs (Remdesivir, Molnupiravir, and Nirmatrelvir) were assessed against the N15 strain in Vero and Calu-3 cells. The inhibition percentage was evaluated at two different time points: 24 and 48 hpi. Remdesivir showed potent antiviral activity against the N15 strain in both Vero and Calu-3 cells with lower IC_50_ values compared to Molnupiravir and Nirmatrelvir. Molnupiravir demonstrated moderate activity, while Nirmatrelvir exhibited strong inhibition but with varying IC_50_ values across different cell lines and time points. These results suggest that Remdesivir may be the most effective among the tested compounds, especially in Vero cells, followed by Nirmatrelvir and Molnupiravir.

## 4 Discussion

Virus isolation is a crucial initial step for various types of research, including genotypic, and phenotypic characterization, as well as the development of preventive and therapeutic products. In this study, one SARS-CoV-2 strain was isolated from 21 saliva/nasal swabs collected from pediatric patients aged from 4.7 to 14.7 years old (Table 1). All samples were collected during the patients exhibited clinical symptoms as well as high virus load (average Ct value of 22.16, Table 1). Based on previous knowledge (Wolfel et al., 2020), these samples were predicted to have a high proportion of positive cultures; however, the actual rate of positive cultures was low. The extended storage duration of these samples, which exceeded 3 years, is believed to be the primary reason for the significantly reduced isolation rate. In addition, the use of Vero cells lacking TMPRSS2 (serine protease) expression, as well as the presence of some clinical samples with relatively high Ct values, may have further reduced the efficiency of virus isolation (Kinoshita et al., 2024). Regarding the *in vitro* properties, the N15 isolate induced CPE in Vero cells, characterized by dead cells clustering into grape-like aggregates. Additionally, viral particles with a spherical shape were observed clustered within membrane-bound vesicles in the cytoplasm (Figure 1). These findings are consistent with previous publications (Goldsmith et al., 2004; Caly et al., 2020; Harcourt et al., 2020; Barreto-Vieira et al., 2022) and imply an active replication of SARS-CoV-2 in cell culture.

Of the genetic characterization, the N15 isolate retains the ancestral D614 residue, similar to the original Wuhan-Hu-1 strain. In contrast, the D614G mutation, located near the C-terminus of the S1 subunit, has been consistently observed in dominant VOCs throughout the pandemic, significantly altering the spike protein’s structure and function to enhance infectivity and transmissibility (Bhattacharya et al., 2021). Moreover, N15 lacks widely recognized, advantageous spike protein mutations found in VOCs—those associated with increased transmission (N501Y, A701V, H655Y), escape from neutralizing antibodies (K417N, E484K/A, N440K, Q493R, and Q498R), and enhanced fusion and cellular entry (P681R) (Sigal et al., 2025). Instead, the N15 strain harbors the N709S mutation near the HR1 (heptad repeat 1) domain in the S2 subunit (Olmedillas et al., 2025). Importantly, the N709S mutation disrupts the N-X-S/T (Asn-X-Ser/Thr) glycosylation motif, which may lead to dynamic conformational changes in the spike protein (particularly affecting RBD open/close) by N-glycosylation loss (Kumar et al., 2023; Pegg et al., 2024).

In the absence of antibody-mediated pressure, the rare amino acids at positions 709 (S) and 614 (D) in the spike protein of the N15 isolate do not affect viral replication *in vitro*, as the replication kinetics of this strain were comparable to those of other SARS-CoV-2 lineages, B.1 and B.1.1.529 (Figure 3). However, under real-world selective pressures occurring in immunized populations, variants with similar genomic profiles are likely constrained by a reduced capacity for immune evasion, explaining their restricted spread and eventual displacement (Figure 4). These rare mutations, together with the limited spread of related variants, highlight important genetic constraints on SARS-CoV-2 evolution. Nevertheless, these rare mutations were not entirely detrimental, as sequences belonging to this lineage were occasionally detected (Figure 4) and persisted at very low frequencies at least until July 2023. A thorough explanation is not yet known, but alternative explanations, such as prolonged replication in immunocompromised individuals (Avanzato et al., 2020), undetected transmission within under-immunized populations, or greater stability of the ancestral lineage of SARS-CoV-2 compared to variants of concern in human biological fluids (Kwon et al., 2023), may account for its unexpected circulation.

Of the antiviral sensitivity, the N15 strain exhibited full susceptibility to Remdesivir, Molnupiravir, and Nirmatrelvir, as indicated in Figure 5. The IC_50_ values observed were largely within the low micromolar to submicromolar range, closely matching those previously reported for the ancestral strain of the Wuhan-like lineage collected in the first stage of the pandemic (Pruijssers et al., 2020; Wang et al., 2020). The similar antiviral response between N15 and the ancestral Wuhan-like strain suggests that N15 retains susceptibility to these direct-acting antivirals, despite being detected in late 2021, a period dominated by Delta variant viruses (Kim et al., 2023). This preserved susceptibility is supported by the absence of mutations in the nsp5 and nsp12 proteins of the N15 strain (Table 2), which are key determinants of antiviral drug susceptibility (European Centre for Disease Prevention and Control, 2023).

Since focusing on the biologic and genetic characterization of a single isolate, this study contained some limitations, such as not including reference ancestral strain of Wuhan-Hu-1 in the assay of viral replication and antiviral drug test. These will be addressed in the future study. Despite of this, bearing a rare mutations N709S in the spike protein and T11M in the E protein, the N15 strain might provide valuable material for comparative studies with current circulating variants. For example, the study of molecular evolution (understanding viral evolution and diversification), and the experimental studies aimed at identifying key genome mutations that may be associated with the biological properties of SARS-CoV-2.

In conclusion, the N15 isolate is closely related to the SARS-CoV-2 Wuhan-Hu-1 reference genome and has replication kinetics comparable to those of lineages B.1 and B.1.1.529. It exhibits unique mutations (N709S in the spike protein and T11M in the E protein) and shows full susceptibility to major antiviral drugs. That strain may serve as preliminary material for future comparative studies, and highlight the need for further investigation to confirm its relevance.

## Data Availability

The datasets presented in this study can be found in online repositories. The names of the repository/repositories and accession number(s) can be found below: https://www.ncbi.nlm.nih.gov/genbank/, PP915627.

## Footnotes

1
https://www.medchemexpress.com

2
https://gisaid.org/database-features/covsurver-mutations-app/

## Supplementary material

Supplementary Figure 1 Phylogenetic tree of the SARS-CoV-2 N15 strain using Nextclade analysis. The complete genome sequence of the N15 isolate (PP195527) derived from a pediatric patient in South Korea is shown on a global phylogenetic tree generated using Nextclade. The x-axis represents the number of mutations relative to the Wuhan-Hu-1 reference genome (NC_045512.2), while the y-axis depicts the Nextstrain clades and their distribution. The N15 strain is highlighted and positioned within clade 19B, indicating a relatively early lineage compared to more recent variants of concern (VOCs) such as Delta (21J), Omicron (21K, 21L), and subsequent Omicron sub-lineages (e.g., 22A–22E, 23A–23H, 24A–24H).
