# Correction: Isolation of a SARS-CoV-2 strain from pediatric patients in South Korea: biologic and genetic characterization

**DOI:** 10.3389/fmicb.2025.1694129

**Published:** 2025-09-09

**Authors:** Hee Chun Chung, Sung Jae Kim, Su Jin Hwang, Sung Hoon Park, Kyoung Min Park, Hyeon Woo Chung, Si Hwan Ko, Dong il Park, Jun-Yeop Shim, Van Giap Nguyen, Jae Myun Lee

**Affiliations:** ^1^Department of Microbiology and Immunology, Institute for Immunology and Immunological Diseases, Yonsei University College of Medicine, Seoul, Republic of Korea; ^2^Department of Companion Animal Health, Kyungbok University, Namyangju, Republic of Korea; ^3^Department of Microbiology and Immunology, Institute for Immunology and Immunological Diseases, Brain Korea 21 Project for Medical Science, Yonsei University College of Medicine, Seoul, Republic of Korea; ^4^R&F Chemical Co., Ltd, Hanam, Gyeonggi, Republic of Korea; ^5^Department of Veterinary Microbiology-Infectious Diseases, Faculty of Veterinary Medicine, Vietnam National University of Agriculture, Hanoi, Vietnam

**Keywords:** SARS-CoV-2, pediatric patient, biology, genetic characterization, N15 strain

In the published article, there was a mistake in the **Funding** statement. The **Funding** statement was originally displayed as:

“This work was supported by the Korea Science and Engineering Foundation (KOSEF) grant (No. 2020R1I1A1A01054539) and by the Korea Research Foundation Grant funded by the Korean Government (NFR-2019R1A6A1A03032869).”

The correct statement is:

“The author(s) declare that financial support was received for the research and/or publication of this article. This work was supported by the National Research Foundation of Korea (NRF) grant funded by the Korean Government (RS-2022-NR067483 and RS-2019-NR040072).”

The original version of this article has been updated.

