# Supplementary material for: Isolation of a SARS-CoV-2 strain from pediatric patients in South Korea: biologic and genetic characterization
Source: Front Microbiol. 2025 Aug 26;16:1654224. doi: 10.3389/fmicb.2025.1654224 (PMC12417416; doi:10.3389/fmicb.2025.1654224)
Supplement: Supplementary file 1 [file Table_1.docx]

**Supplementary Table 1.** List of primers used in this study (developed by OPTOLANE Technologies Inc, Korea).

| **Primer**  **names** | **Detection method** | **Sequence (5’-3’)** | **Target Gene** | **Location (nt)** | **Purpose** | **Size**  **(bp)** |
| --- | --- | --- | --- | --- | --- | --- |
| SARS-CoV2F-356 | Digital PCR  Real-time PCR | CTGGACTTCCCTATGGTGCT | Nucleocapsid | 356-375 | Quantification | 91 |
| SARS-CoV2R-446 |  | CGGGTGCCAATGTGATCTTT |  | 427-446 |  |  |
| SARS-CoV2 Probe-394-415 |  | FAM-AGGCTCCCTCAGTTGCAACC-BHQ1 |  | 394-415 |  |  |
