# Supplementary figures and images for: Isolation of a SARS-CoV-2 strain from pediatric patients in South Korea: biologic and genetic characterization
Source: Front Microbiol. 2025 Aug 26;16:1654224. doi: 10.3389/fmicb.2025.1654224 (PMC12417416; doi:10.3389/fmicb.2025.1654224)

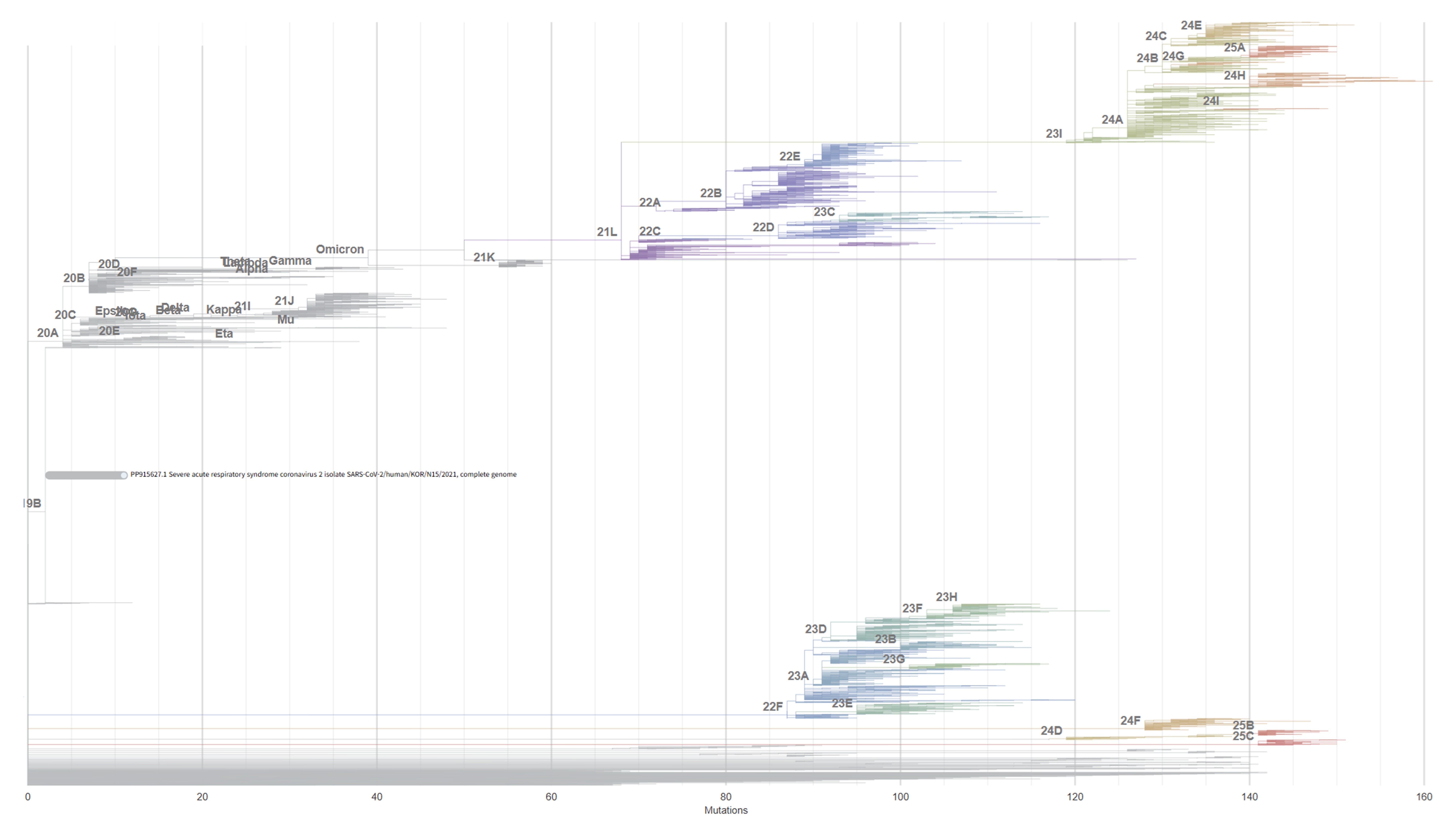

Supplement: Supplementary Figure 1 — Phylogenetic tree of the SARS-CoV-2 N15 strain using Nextclade analysis. The complete genome sequence of the N15 isolate (PP195527) derived from a pediatric patient in South Korea is shown on a global phylogenetic tree generated using Nextclade. The x-axis represents the number of mutations relative to the Wuhan-Hu-1 reference genome (NC_045512.2), while the y-axis depicts the Nextstrain clades and their distribution. The N15 strain is highlighted and positioned within clade 19B, indicating a relatively early lineage compared to more recent variants of concern (VOCs) such as Delta (21J), Omicron (21K, 21L), and subsequent Omicron sub-lineages (e.g., 22A–22E, 23A–23H, 24A–24H). [file Image_1.jpeg]
